# Econeurobiology and brain development in children: key factors affecting development, behavioral outcomes, and school interventions

**DOI:** 10.3389/fpubh.2024.1376075

**Published:** 2024-09-26

**Authors:** Raed Mualem, Leon Morales-Quezada, Rania Hussein Farraj, Shir Shance, Dana Hodaya Bernshtein, Sapir Cohen, Loay Mualem, Niven Salem, Rivka Riki Yehuda, Yusra Zbedat, Igor Waksman, Seema Biswas

**Affiliations:** ^1^Department of Natural and Environmental Sciences, Faculty of Education, Oranim Academic College, Kiryat Tiv'on, Israel; ^2^The Institute for Brain and Rehabilitation Sciences, Nazareth, Israel; ^3^Econeurobiology Research Group, Research Authority, Oranim Academic College, Kiryat Tiv'on, Israel; ^4^Ramat Zevulun High School, Ibtin, Israel; ^5^Department of Physical Medicine and Rehabilitation, Harvard Medical School and Spaulding Rehabilitation Hospital, Boston, MA, United States; ^6^Department of Computer Science, Haifa University, Haifa, Israel; ^7^Bar Ilan University Medical School, Tzfat, Israel; ^8^Global Health Research Laboratory, Department of Surgery B, Galilee Medical Center, Nahariya, Israel

**Keywords:** public health, brain development, brain connectivity, plasticity, learning, education, self-regulation, social determinants of health

## Abstract

The Econeurobiology of the brain describes the environment in which an individual’s brain develops. This paper explores the complex neural mechanisms that support and evaluate enrichment at various stages of development, providing an overview of how they contribute to plasticity and enhancement of both achievement and health. It explores the deep benefits of enrichment and contrasts them with the negative effects of trauma and stress on brain development. In addition, the paper strongly emphasizes the integration of Gardner’s intelligence types into the school curriculum environment. It emphasizes the importance of linking various intelligence traits to educational strategies to ensure a holistic approach to cognitive development. In the field of Econeurobiology, this work explains the central role of the environment in shaping the development of the brain. It examines brain connections and plasticity and reveals the impact of certain environmental factors on brain development in early and mid-childhood. In particular, the six key factors highlighted are an environment of support, nutrition, physical activity, music, sleep, and cognitive strategies, highlighting their potential to improve cognitive abilities, memory, learning, self-regulation, and social and emotional development. This paper also investigates the social determinants of health and education in the context of Econeurobiology. It emphasizes the transformative power of education in society, especially in vulnerable communities facing global challenges in accessing quality education.

## 1 Introduction

The human mind is capable of astonishing function. We are still understanding how the brain develops and adapts to life experiences and the environment in which a child grows. A glance at the United Nations Sustainable Development Goals (1), shows the importance of a child’s environment in maximizing educational opportunity and attainment. The first 3 years of life are when the brain is most plastic (capable to change and adapt through life experiences). Research has shown that early environmental influences significantly shape brain architecture. For example, studies have demonstrated how socioeconomic factors can affect brain development, linking poverty to alterations in brain structure and function (2, 3).

This article focuses on brain research related to child development and learning in early and middle childhood (2–5 years and 6–11 years, respectively), examining how environmental and epigenetic factors – collectively referred to as the econeurobiology of the brain – interact to influence brain development. We present an econeurobiological model that integrates these factors and offers educational and policy proposals inspired by Gardner’s model of intelligence. This model is first introduced here to provide a framework for understanding the subsequent sections of the manuscript. Additionally, a detailed outline of the manuscript is provided to help readers grasp the overall picture and navigate the complex interactions discussed in the following sections.

Econeurobiology is defined as the study of how environmental and epigenetic factors influence neurobiological developmental processes in the brain, particularly during early childhood. This multidisciplinary field examines the intricate interactions between the environment, genetic predispositions, and brain development.

In order to understand how to optimize learning inside and outside the classroom, an appreciation of how the human brain develops and functions, and how cognition, concentration, learning and memory are enhanced through brain connectivity and plasticity is important. The context in which a child grows is crucial, especially when environmental exposure can affect – positively or negatively – the dynamics behind neural functional connectivity throughout development. The context can enhance learning, well-being, and resilience when environmental conditions are optimized, but can substantially disadvantage a child who is subjected to environments of continuous stress and privation (2–4). These factors exert their effects into adulthood, with implications for individuals, families, communities, and societies. These factors are both the social determinants of learning and the social determinants of health. Their interaction is at multiple levels and is cumulative. Thus, positive interventions that affect health and education in early childhood have potentially profound and far-reaching impacts on individuals, families, and their communities (5–8).

Drawing on a wide range of literature from neurodevelopmental biology to pedagogy, this paper introduces the econeurobiological model, which examines how environmental and epigenetic factors influence neurodevelopmental processes in the brain. Our model integrates these factors to understand their impact on brain development, especially in children. In addition to detailing the neurobiological mechanisms involved, the paper presents educational and policy proposals inspired by Gardner’s model of intelligence, aiming to enhance cognitive development through tailored educational strategies.

### 1.1 Evolution of the human brain

**Figure 1:**
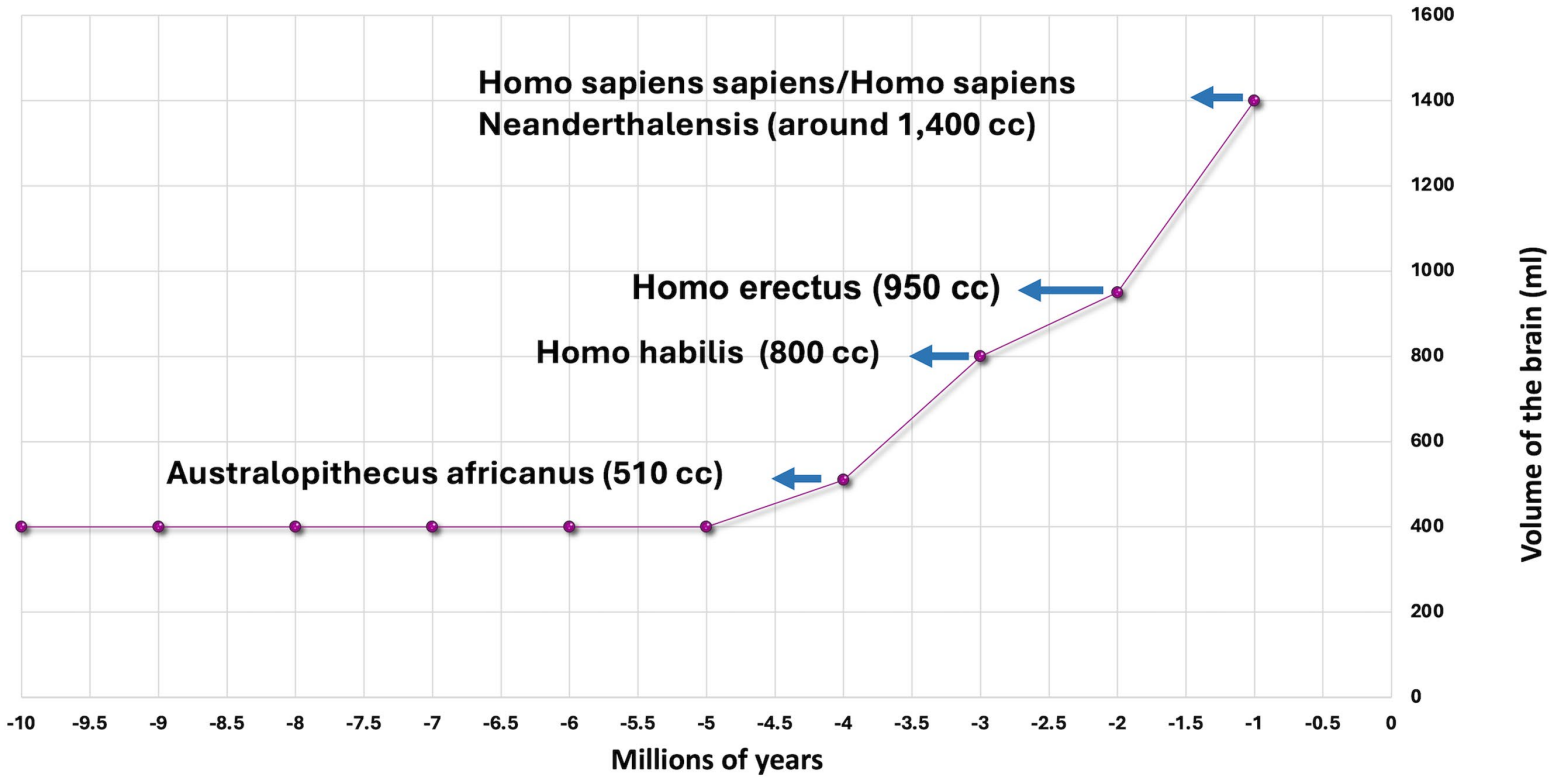
The increase in brain volume among primates.

Research into the evolution of primates has revealed that the uniqueness of the human brain is not determined by its volume but by the number of neurons within the brain and the network of connections between them (9). Human brain volume as a ratio to body mass is larger than that of other mammals. For example, the adult elephant brain weighs 4–5 kg, while the human brain weighs 1.5 kg (1.5 liters by volume). The human brain’s 86 billion neurons contrast with a gorilla’s 30 billion. A gorilla needs to eat for about 8 h a day in order to meet the energy requirements of its brain. Humans need to eat substantially less (9, 10). The evolution of human brain size is probably a function of evolutionary changes in diet, foraging for food, and optimizing energy metabolism. One explanation is the invention of fire and the consumption of cooked food – food efficiency (10). Homo erectus (the upright man) first began using fire in areas of South Africa and present-day Kenya about 1.5 million years ago (11). Beyond the impact of cooking on brain architecture, it is likely that environmental pressures and social competition have played a significant role in brain volume development and an increase in the number of neurons (12). Cooked food is more easily digested and yields more calories than raw food (13). The human brain makes up 2% of body mass, but it consumes about 20% of daily energy, which is linearly related to the number of neurons and the quality of neural connections. The energy consumption of a neuron is constant and does not depend on the size of the brain (14). Figure 1 illustrates the evolutionary increase in brain volume.

**Figure 2:**
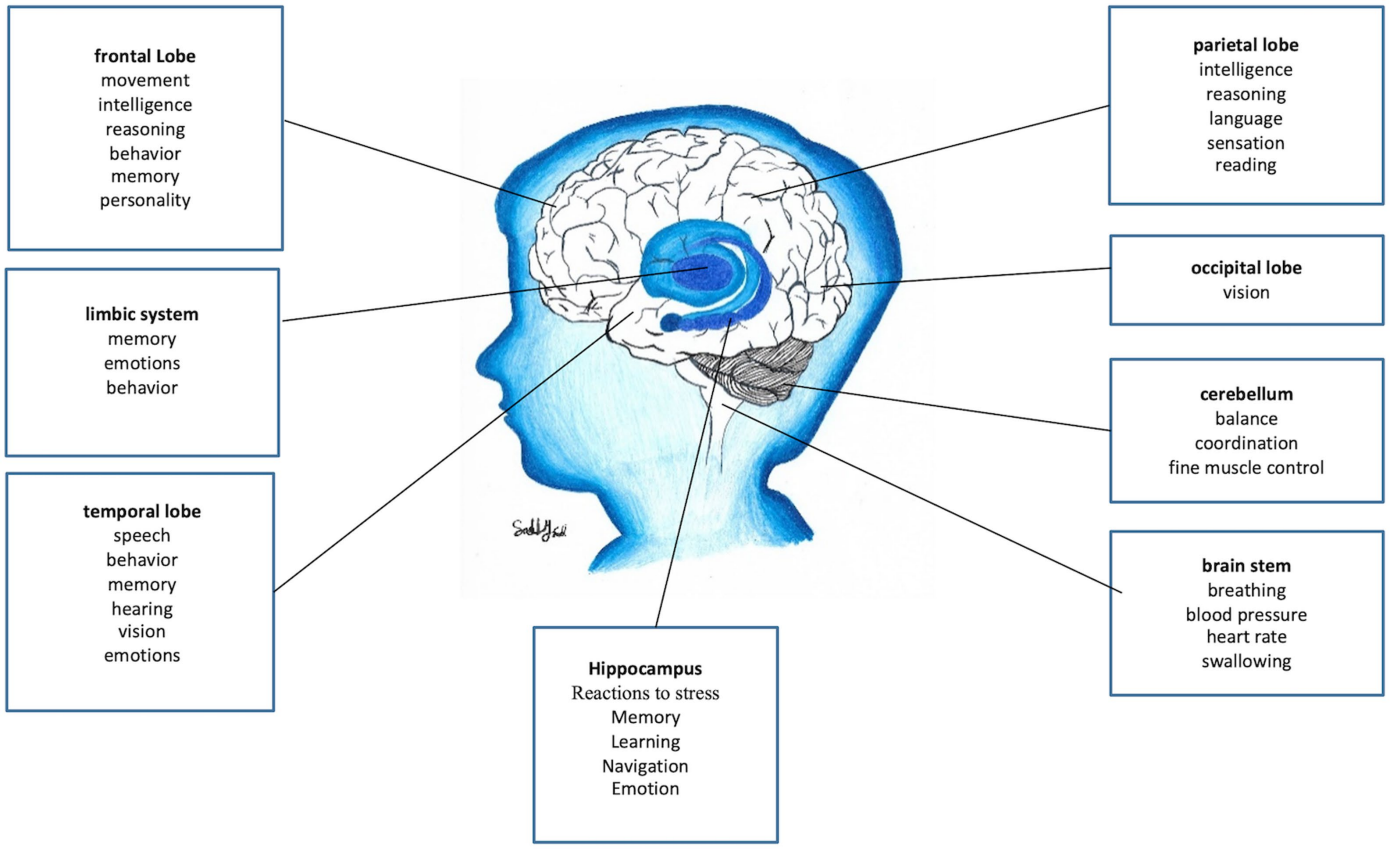
Overview of brain functions. This simplified summary highlights major brain regions and their primary functions. Note that many functions involve interactions across multiple areas. Key regions include the hypothalamus, which has a major role in reactions to stress (15), brainstem (pain modulation and sympathetic outflow), and the reward system (VTA, nucleus accumbens, prefrontal cortex) (figure drawn by Sally Saadi).

The expansion of the neocortex through primate evolution parallels the greater cognitive capacity of the human brain (Figure 2). Neuroanatomical experiments have shown that exercise can increase dendrites, spines, and other structures, indicating that functional activity drives the anatomical reshuffling of neurons and areas. For example, motor training induces experience-specific patterns of plasticity across the motor cortex and spinal cord (16). Humans have the largest frontal cortex of all primates and the entire cerebral cortex of humans in proportion to body size is larger than in other primates (9, 10, 13, 16). It is human fetal development of the neocortex and subsequent cellular organization and connectivity between brain areas that distinguishes it from the brains of other primates in terms of intellectual capacity (17).

### 1.2 Building brain architecture

During the first years of our lives, over a million new neural connections are formed every second. This rapid rate of synapse formation highlights the high brain plasticity during early childhood, which is crucial for significant learning potential. The rate of connection formation and brain plasticity decreases with age (18).

The stages of brain development include neurogenesis, cell migration, differentiation, maturation, synaptogenesis, cell death and pruning, and myelogenesis. Neurogenesis begins in the early embryonic stage and is usually completed 5 months after birth. Neurogenesis in the hippocampus, however, continues through life, not only in the hippocampus but also in the subventricular zones, rostral migratory stream, and olfactory bulb network, playing a crucial role in learning and memory (19).

Each of the stages of development is affected by neurohormonal and environmental factors. Dendrites in babies protrude from the cell body and extend dynamically over the first 2 years of life. Axons grow much faster than dendrites and, therefore, through contact with the dendrites of other neurons, influence dendrite differentiation and neuronal connectivity. An increased and redundant number of neurons and connections are formed in the first 2 years – more than needed; thus, cell death and synaptic pruning take place. After a period of rapid proliferation and the construction of neural pathways and networks, neural connections are reduced through pruning so that electrical circuits in the brain can operate more efficiently (19).

Experiences in early childhood - the critical period – determine the process of neurological development and the architecture of neural networks – the wiring in the brain (6, 20, 21). Networks that are continuously used are strengthened while unused networks are pruned (22, 23). Every active thought, feeling, or behavior leads to the activation of thousands of neurons that connect together. Repetitive patterns of behavior or thought result in automatic neural activation. On the other hand, patterns of behavior and thought that are suppressed or interfere with established network formation disappear. The creation of environments that stimulate neural networks that support learning is crucial. Equally, shielding children from negative environmental factors substantially impacts cerebral plasticity (24). Thus, a child’s first years significantly affect the architecture of the continually developing and changing brain as the child’s experiences shape the formation and pruning of connections through the process of developmental synaptogenesis (6, 25, 26). A key neurotransmitter implicated in embryonic development is gamma-aminobutyric acid (GABA), controlling cell migration. Glutamate and glycine receptors appear from the first phases of cortical development, and while a detailed study of neurotransmitters is beyond the scope of this article, interference with neurotransmitter signaling has been implicated in several neurodevelopmental disorders (27–29). Dysregulation in neuronal differentiation and signaling with abnormal synaptic function are similarly associated with dopaminergic (and other neurotransmitter) pathways in early brain development implicated in cognitive, behavioral, and psychiatric disorders.

**Figure 3:**
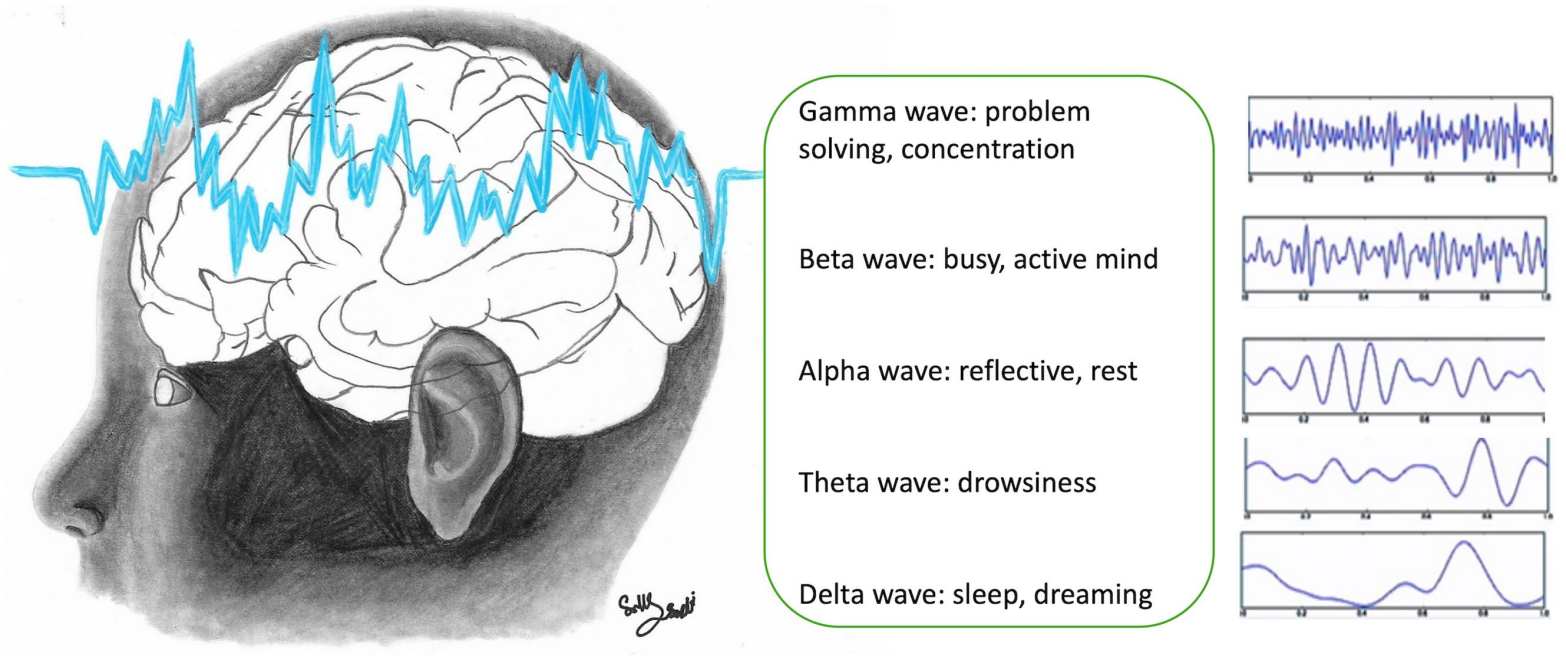
Electroencephalogram (EEG) waves in the brain (figure drawn by Sally Saadi).

Impaired connectivity between neurons has an immediate effect on cognitive, behavioral, and emotional function, which, in turn, affect learning (5, 6), and emotional regulation (30). When an individual vocalizes a word after reading, distinct neural networks are activated compared to when the word is spoken after being heard. This difference is highlighted in studies utilizing functional MRI (fMRI) and electrophysiological measurements, which show that reading and auditory processing engage different cortical areas, reflecting the various pathways involved in language processing and cognitive functions (6, 31, 32). The stimulus and subsequent processing of information are different. Thus, several stimuli modify cortical organization and connectivity in specific and different ways. Understanding which parts of the cortex are involved in a process is complex but functional MRI (fMRI) and electrophysiological measurements of cortical oscillations are of value. Electrophysiological analysis involves the study of types of brain electrical activity and their intensity and distribution over the cortical surface. In fact, EEG has been used to evaluate brain maturity from newborns to infants, where specific patterns of activity such as the occurrence of posterior rhythm, sleep spindles, and vertex waves represent markers of adequate electrographical development (especially when these events occur between 6 and 8 weeks after birth) (31). Some neural correlates of behavior and cognition can be associated with EEG activity. EEG activity across different frequency bands is associated with a variety of cognitive and physiological states, but these associations are complex and multifaceted. For instance, gamma waves are often linked to cognitive processing and problem-solving activities. Beta waves are associated with active thinking and focus, while alpha waves are related to relaxation and calm states. Theta waves are typically observed during meditative, drowsy, or creative states, and delta waves, while prominently associated with deep sleep, can also appear during focused attention and certain cognitive tasks (Figure 3) (5–8, 33, 34). Thus, it should be possible to see whether a child is calmly learning by monitoring electroencephalography (EEG) activity. In contrast, previous studies of children exposed to toxic stress showed patterns of neural activity that reflect cortical hypoactivation, including reduced alpha power with increase power of the theta band (35). Therefore, EEG can be a valuable tool in evaluating neurophysiological markers of brain maturity in the context of cognitive and behavioral function. Recent advances in signal analysis methods have been used to generate predictive models, where cognitive processing, emotional states, or behavioral performance are correlated with biological markers of EEG activity, to monitor cognitive behavioral development in children or to assess responses to therapeutic interventions.

### 1.3 Developmental plasticity

Most of the human behavior result from social interactions and exposure to the environment. Some, however, are found to be prewired into the brain, such as the capacity to develop language. Similarly, the brain’s ability to process and integrate visual stimuli exist almost immediately after birth (36). Nevertheless, the expansion of brain development is further boosted as the newborn is exposed to new sensorial and emotional experiences. It is during this developmental stage that neural networks, primed to receive new stimuli, compete for survival by becoming more efficient and precise in response to environmental demands. The neuronal mechanisms supporting the formation of new memories and learning consist of use-dependent long-term modifications of synaptic transmission. Moreover, this synaptic connectivity must be strengthened by repeated temporal firing to promote long-term potentiation (LTP) (37). It has been confirmed that tetanic stimulation of excitatory pathways led to long-lasting enhancement of the efficacy of the synapses between the activated fibers and the respective postsynaptic neurons (38), this increase in synaptic efficacy occurs only if the postsynaptic neurons respond by generating action potentials to the ongoing tetanic stimuli, thus fulfilling the criterion of contingent pre and post synaptic activation (39). When postsynaptic neurons are prevented to respond to the stimuli, synaptic strengthening is void, leading to a decrease in synaptic efficacy, this phenomenon is known as long-term depression (LTD). The molecular basis supporting the generation of LTP and LTD are beyond the scope of this chapter, yet, it has been shown these synaptic modifications are calcium dependent and that the polarity of the modifications depends on the rate of rise, and the amplitude of this calcium increase (40). Thus, fast, and strong intracellular increase of calcium lead to LTP, while slow and smaller increases trigger LTD (41). Therefore, stimulus-induced, and self-generated neuronal synchronized activity, plays a crucial role in the activity-dependent shaping of the neuronal architecture during development. This has a direct impact in the formation of cognitive skills in humans, as LTP/LTD activity must be finely tuned to promote synaptic strengthening and synaptic consolidation during learning experiences.

Hence, endogenous and exogenous factors influence how neuronal circuits develop early in life. Exposure to a nurturing environment can further facilitate neuronal growth and refinement, while exposure to adverse conditions will have a detrimental effect.

Consequently, families must be supported by their societies to provide the most favorable – econeurobiology – environment for development, so children can have the opportunity to achieve their physical and intellectual potential.

### 1.4 The capacity for learning

Understanding an individual’s cognitive abilities, temperament and behavior depends on an understanding of neuroscience and neurological function (33). The brain contains about 86 billion neurons, 16 billion of which are in the cerebral cortex. This number of neurons is unique to humans and explains the high-energy requirements of the human brain compared to other living creatures. The human brain constantly changes and renews itself in response to new experiences, knowledge, and information from the environment – brain plasticity (7, 8, 10). Neurons are the basic units of information processing and decision-making. The large number of neurons in the human cerebral cortex and neural networks are responsible for brain connectivity and higher functioning (10, 13). Neurons connect with each other through a process called synaptogenesis, with electrical transmission resulting in the secretion of neurotransmitters. One neuron may form as many as 10,000 new connections (8, 10, 13). It is the formation of these connections that is crucial to the process of learning. Plasticity allows people to learn and adapt. Thus, while at birth, all individuals have approximately 86 billion neurons, the environment and an individual’s social interactions shape the structure and architecture of the brain and facilitate cognitive and learning processes. These processes include the formation of ideas, the solution of multivariate problems, strategies to navigate daily activities, contingency planning, and the expression of character, emotion, and behavior. The capacity for learning and self-regulation (moderation of one’s behavior) are essential to resilience in childhood and adult life. It is the processing of information in the cerebral cortex and connectivity, especially with the limbic system, that makes it possible for an individual to weigh information, draw conclusions, judge good and evil, remember events and their significance, learn from mistakes, plan ahead and change these plans as circumstances change, and form patterns of behavior and personality. The organization of the cerebral cortex is dependent on the individual’s exposure to environmental factors and personal life experiences that affect gene expression; thus, the environment of a child at home and in school influences the creation of neuroproteins and transmitters that promote brain connectivity (42). The more frequently a process of experience takes place, the stronger the connections between neurons. Conversely, a failure to stimulate a child and the withholding of affection diminish connectivity and result in the loss of neurons, a reduced capacity to learn, and an inability to self-regulate emotions and behavior. Even in childhood, provided children are loved and supported, the brain is shaped by challenges, adversity and failures, as well as successes. This builds resilience and the capacity to learn and adapt.

Neural pathways in cognition describe the complex interaction between various brain regions, including the limbic system, the prefrontal cortex, and the reward system. The limbic system, traditionally associated with emotions and memory, plays a significant role in both conscious and unconscious processing, influencing behaviors that range from instinctive to deliberate. The prefrontal cortex, on the other hand, is linked to reasoning and analytical thinking. Kahneman’s dual-process theory categorizes these functions into ‘fast’ and ‘slow’ systems, but this dichotomy remains a topic of debate in cognitive psychology and neuroscience, with emerging research suggesting more integrated and overlapping roles of these systems (43).

**Figure 4:**
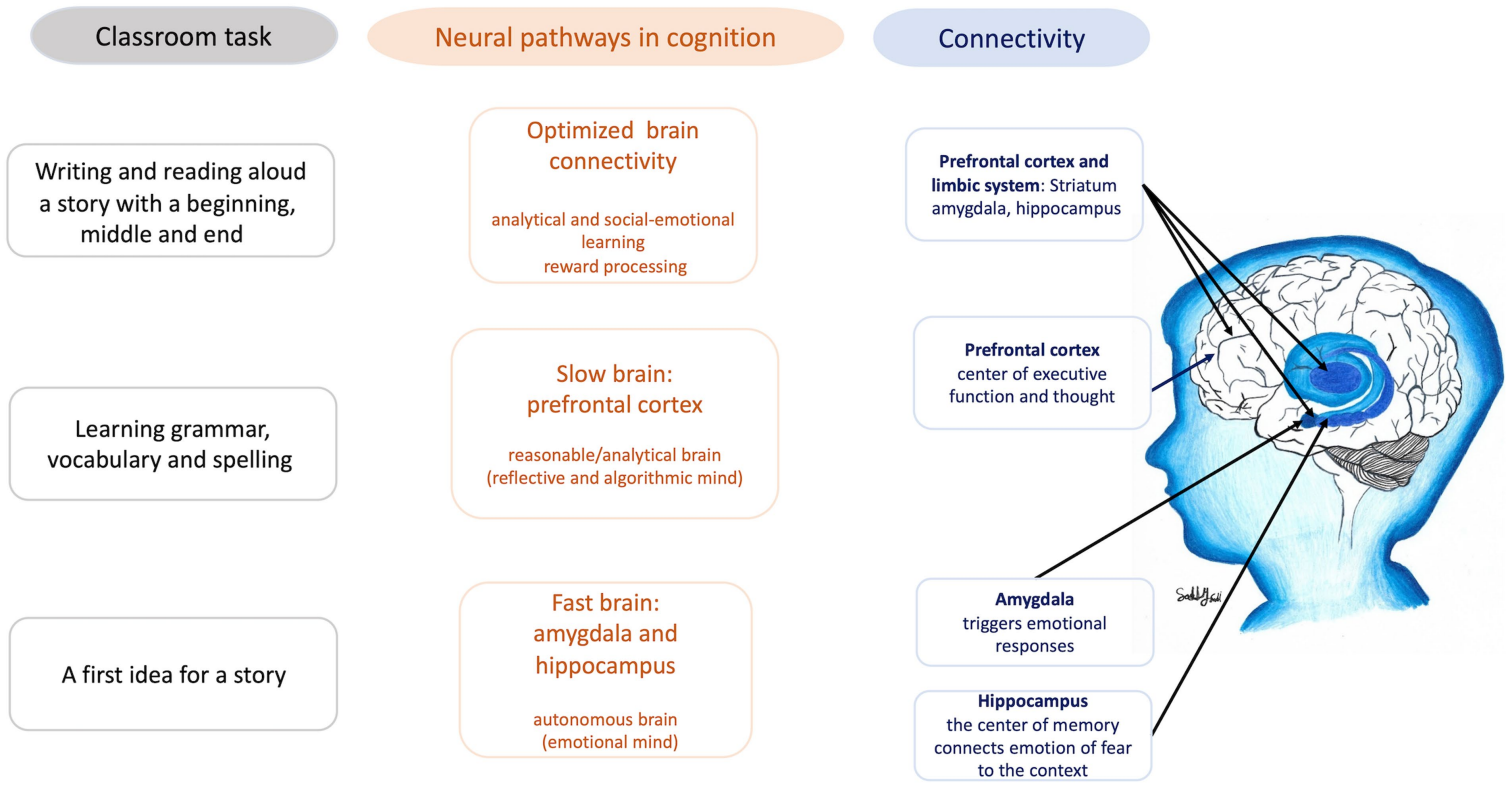
Connectivity between areas of the brain during a typical classroom task (figure drawn by Sally Saadi).

Both pathways are crucial to effective learning in the classroom, influencing concentration, focus, memory, and the evaluation of what is learned. Figure 4 illustrates how these neural pathways influence classroom learning, using the example of writing a story. The optimal development of connectivity between these systems is essential for fostering both quick, intuitive thought and slower, more considered analysis, contributing to what is termed the ‘optimized brain.’ Creating conditions that support this connectivity is vital for developing resilience and adaptive capacities in children, enabling them to manage adversity more effectively. Furthermore, the ‘reward’ pathways of the brain, primarily mediated by dopamine produced in the ventral tegmental area (VTA) of the midbrain, connect with the limbic system, including the nucleus accumbens, amygdala, and hippocampus. Engaging in pleasurable learning activities, music, and aerobic exercise, alongside receiving positive feedback and praise, activates these motivation and reward pathways, driving the formation of memories, social–emotional learning, and behaviors essential for effective learning and socialization (5, 6, 10, 44).

The interaction of these brain systems forms a complex and integrated network essential for learning and behavior regulation. The reward system, which includes the VTA, nucleus accumbens, amygdala, hippocampus, hypothalamus, and striatum, plays a crucial role in regulating motivation toward specific objects, persons, or actions. Motivation is vital for learning and other functions, as it drives the engagement and persistence necessary for acquiring new skills and knowledge. The VTA connects with the limbic system and nucleus accumbens, which are in turn connected to the amygdala, hippocampus, hypothalamus, and striatum. These interconnected networks form a comprehensive system for behavior regulation, linking different hubs from the prefrontal cortex to the brainstem. This underscores the importance of a balanced and well-connected neural network for optimal cognitive and behavioral functioning.

This article reviews the evolution of human brain development and explains the conditions for optimal brain architecture, plasticity, and childhood learning. The influence of environmental factors – econeurobiology – is discussed in the context of the social determinants of health and their influence on intellectual potential.

## 2 Environmental enrichments at different developmental stages

Research has consistently shown that environmental enrichment at different developmental stages positively influences brain plasticity, leading to improvements in health and achievement. Studies reveal that both physical and social enrichment can cause functional, structural, and molecular changes in the brain, such as increased growth factor expression and neurogenesis (45, 46). This is evident in investigations of early environmental enrichment in rats. Environmental enrichment in these studies typically involves providing animals with a stimulating environment that includes a variety of objects, opportunities for physical activity, and social interactions. Such enrichment has been shown to influence brain development and function by enhancing neuroplasticity and promoting the expression of growth factors. For instance, these studies highlight sex-specific responses in oxytocin (OT) and brain-derived neurotrophic factor (BDNF) expression (45, 46). For instance, while physical enrichment enhances motor and cognitive functions and hippocampal BDNF expression in both sexes, combined physical and social enrichment is particularly beneficial for females. This suggests an OT-based mechanism that selectively stimulates BDNF response in a region-specific manner, depending on the type of enrichment (45).

Further studies in male mice post-weaning indicate that environmental enrichment increases social behavior, moderates stress-related physiological markers, and boosts BDNF levels in the prefrontal cortex. Conversely, removing female rats from enriched environments leads to behaviors indicative of psychiatric disorders, such as increased passive coping and hyperphagia, along with signs of HPA axis dysregulation (46). These findings underscore the potential of environmental enrichment in early life to affect parental care and offspring outcomes, possibly extending to transgenerational effects. However, translating these paradigms from animal models to clinical settings, such as in stroke patients, requires more alignment for effective implementation. Environmental enrichment shows promise for a wide range of neurological and psychiatric conditions (46).

In terms of aging, environmental enrichment (EE), even without exercise, can prevent cognitive decline and reduce age-related brain deterioration. This is particularly significant for populations where physical exercise is impractical. EE alone has been found to reduce anxiety, enhance memory, and potentially be more effective in older animals. This suggests that EE can mitigate cognitive loss with age independently of physical activity (47).

Moreover, EE enhances performance in various behavioral tasks, like spatial memory and anxiety-related behaviors in adult Wistar rats (48). While EE reduces anxiety and improves spatial memory accuracy, its impact on attentional tasks is less pronounced. Notably, EE also affects brain functional networks, promoting more efficient connectivity (49).

Lastly, a comprehensive review of 375 studies, focusing on 142 of higher quality, reveals the significance of non-cognitive skills acquired early in life on later outcomes. These skills show consistent effects on academic achievement, psychosocial, language, and cognitive outcomes. The findings highlight the need for better study design and reporting, especially in randomized controlled trials and observational studies. Interventions targeting the development of non-cognitive skills could be particularly beneficial for disadvantaged children, suggesting a broader societal impact (49). Altogether, these findings show how dramatic the influence exerted by the environment can be on brain plasticity. Studies using the EE paradigms have indicated several molecular mechanisms that might emerge as possible ways of accession for a successful treatment of neuropathological conditions affecting the juvenile and adult CNS (50). These studies also reveal how EE influence cognitive development as everyday experiences can potentially enhance or inhibit cognitive plasticity and therefore the ability to learn (51).

In summary, environmental enrichment at various developmental stages offers profound benefits for brain plasticity, ultimately enhancing achievement and health outcomes.

## 3 The negative impact of trauma and toxic stress on plasticity

Toxic stress, as observed in children’s brains, is characterized by an adverse response to early life challenges and can exert far-reaching negative effects on physical, psychological, and behavioral well-being (52). This type of stress can result in persistent alterations to the brain’s stress response systems, which may compromise an individual’s ability to manage stress and regulate emotions in the future (53). The implications of toxic stress extend to epigenetic modifications, potentially leading to enduring alterations in gene expression and subsequent child development (54). Furthermore, the family setting plays a critical role, with the implementation of physical punishment by parents being identified as a significant source of toxic stress that can impact brain architecture and function (55).

Adverse childhood experiences (ACEs) – that generate toxic stress – are defined as traumatic events that occur before the age of 18 years that can have major consequences for behavioral, cognitive, and physiological development affecting one’s life-course health trajectory (56) ACEs can include maltreatment, severe household dysfunctions, the loss of one or both parents for any reason, and other events such as severe bullying, natural disasters, extreme poverty, or exposure to warfare. These traumatic experiences elicit strong physiological stress responses that prepare the body to face dangers or hazards, conditioning it into a fight, flight, or freeze mode (57).

Emotional trauma can profoundly affect brain plasticity by altering neuronal circuits and synaptic connections (58). The stress induced by trauma typically activates the neural systems related to attention and memory, which leads to a temporary increase in synaptic plasticity within the hippocampus (59). Initially, this response may enhance memory, but over time, the hippocampus often becomes less responsive to new excitatory plastic changes (60). The enduring nature of traumatic memories, particularly those resistant to extinction that are characteristic of posttraumatic stress disorder (PTSD), is likely a consequence of these changes in plasticity (61). Moreover, chronic exposure to ACEs have been linked to inflammation in childhood, adolescence, and across adulthood (62). Chronic inflammation has been established as an overlying mechanism in which the immune system contributes to the development of later disease (63). Cytokines, which coordinate inflammatory processes, are often used as biomarkers to assess levels of inflammation. Children who were exposed to toxic stress between the ages of 6–8 years were found to have higher levels of C-Reactive Protein (CRP) and Interleukin-6 (IL-6) at 10 years. In addition, ACEs prior to 9 years were associated with higher levels of CRP at age 15 (62). The negative impact that chronic inflammation has in a developing brain, particularly during the maturation of cognitive and emotional functioning, may be considered an important factor for the presentation of disease or psychopathology later in life. It has been demonstrated that exposure to psychosocial deprivation early in childhood, is associated with smaller gray and white matter volume and global reductions in cortical thickness (64). Moreover, these structural abnormalities were correlated with impaired cognitive functioning and increased development of psychopathology (65, 66). However, when children are removed from their adverse environment – removed from toxic stress – by placing them in a safe and caring environment, these changes reverse, predominantly in the lateral and medial prefrontal cortex and white matter tracts that connect the prefrontal and parietal cortex (67), which are cortical structures associated with cognition and emotional regulation.

The detrimental impacts of prolonged trauma and stress on brain plasticity are well-established (68–70). Chronic stress can lead to reduced metabolism and synaptic density in the hippocampus and prefrontal cortex, which then necessitates behavioral adaptations (71). Additionally, chronic stress can lead to systemic changes that contribute to allostatic load, but the brain retains a degree of resilience and can react positively to interventions aimed at promoting plasticity and thus aid recovery (72). Research also shows that acute stress can modify inhibitory neurotransmitters, such as gamma-aminobutyric acid (GABA), influencing the stress axis and potentially affecting plasticity (72).

In conclusion, understanding the effects of trauma and stress on neural plasticity is essential for developing therapeutic strategies. These strategies not only aim to foster resilience but also address the persistent adverse effects of stress, thereby contributing to healthier brain function and mitigating the long-term consequences of stress-related disorders.

### 3.1 The catastrophic effects of violent conflicts on econeurobiology

War and violent conflicts often result in devastating consequences, including loss of life, displacement of civilians, destruction of infrastructure, and long-lasting socio-economic impacts. Communities endure trauma, and the conflict can exacerbate existing political, ethnic, or religious tensions, making post-war recovery challenging. Humanitarian crises may arise with limited access to basic necessities, hindering the overall development of the affected regions. Regrettably, war and violent conflicts disproportionally affects the most vulnerable populations, children, women, and the older adult, who in addition to be exposed to death, injury, disabilities, illness, and rape, they also suffer from intense and continuous psychological suffering. Children are exposed to situations of terror and horror that have detrimental effects on neural development and may leave enduring cognitive deficits and psychopathology such as posttraumatic stress disorder (PTSD), depression, and anxiety. Furthermore, these negative effects may be prolonged by exposures to further privations and violence in refugee situations (73).

Although the ideal action would be the complete removal of children from war, and to place them in a supportive and caring environment, the socio-political realities, frequently prevent any moral and/or humanitarian effort to achieve this goal. Therefore, humanitarian organizations are left with limited option to mitigate the terrible consequences of these violent conflicts have on children. The destruction of the econeurobiology must be considered a war crime, as the devastating effects on neural development will adversely affect the life trajectory of those children who survive and prevent them to reach their full potential.

## 4 Education as a determinant of health and social mobility

**Figure 5:**
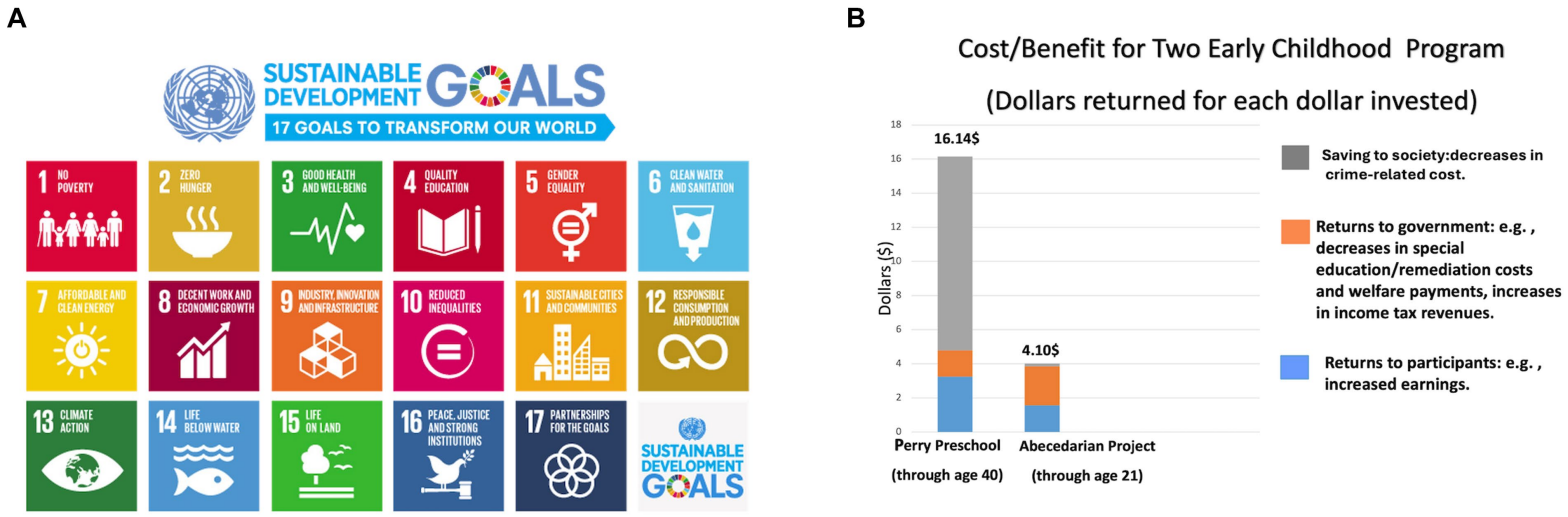
**(A)** Education as a sustainable development goal. Adapted with permission from the illustration: “2030 Sustainable Development Goals.” © Courtesy of the United Nations (1). **(B)** The return on investment of early childhood education. Adapted with permission from the illustration: “Cost/Benefit for Two Early Childhood Programs.” © Center on the Developing Child at Harvard University (5).

The adolescent brain becomes capable of performing more complex functions but loses adaptability in terms of lifestyle change or behavior. This decline in brain plasticity emphasizes the importance of investment in positive learning environments in early childhood. Risk factors for poor academic attainment accumulate well before a child begins school (5, 7). An early nurturing environment where children are exposed to positive interactions and encouraged to learn determine literacy, numeracy, motor skill, cognition, and emotional development in school (5, 7, 74). By the age of 3 years, the children of high-income professionals have been found to have twice the vocabulary of children from low-income families (71). One dollar invested in early childhood yields a benefit of $ 16.14 (Figure 5), while an investment of $ 1 in those over 21 years of age yields only $ 4.10 (5, 8, 23).

This paper acknowledges the significant influence of country and culture on educational theory and practice, particularly in the sections on early childhood programs, elementary curriculum, and STEAM education. The costs and benefits of early childhood programs presented in Figure 5B were estimated primarily for the United States. However, it is crucial to recognize that institutional and economic circumstances vary widely between nations, impacting the applicability and effectiveness of these programs.

Motivation and skill for those engaging with young learners in school are essential. Schools require investment and teachers require training and support. Class sizes should be smaller, and lessons should engage each child, fire the imagination, and allow them to explore what there is to learn about the world within a safe and supportive environment. Extracurricular education and activity are as important as classroom learning (75). Integrating sport, music, art, and activity into classroom lessons and effectively timing lessons, breaks and the school day add to the quality of education, how a child learns, what they learn, what they remember, and how they learn to learn (76–78). While standardized tests of knowledge acquisition and critical thinking (based on Bloom’s taxonomy of critical thinking, which categorizes cognitive skills from basic recall of facts to higher-order thinking skills such as analysis and evaluation) are used to assess the attainment of educational milestones, class attendance, participation, levels of substance abuse, crime, teenage pregnancy, and child employment are important markers of the effects of education as a determinant of health, future employment, economic security, and social mobility. Bloom’s taxonomy classifies educational learning objectives into six hierarchical levels: knowledge, comprehension, application, analysis, synthesis, and evaluation. This framework helps educators structure and evaluate the effectiveness of their teaching by focusing on the development of higher-order cognitive skills (77–79). Poorly performing schools are in themselves a determinant of the failure of a child to meet his or her educational potential and life goals. Funding educational programs that target early school-age children is important but, in reality, the education of the poorest children in society remains inadequately funded and badly managed. Addressing the determinants of poverty are a priority in improving education from early childhood (80). Education remains the single most important factor in lifting children out of poverty (81).

## 5 Econeurobiology: key factors that influence the developing brain

A child’s environment and the social determinants of health influence the biological mechanisms that shape an individual’s cognitive, social, psychological and behavioral development (23).

### 5.1 The nurturing and loving environment

**Figure 6:**
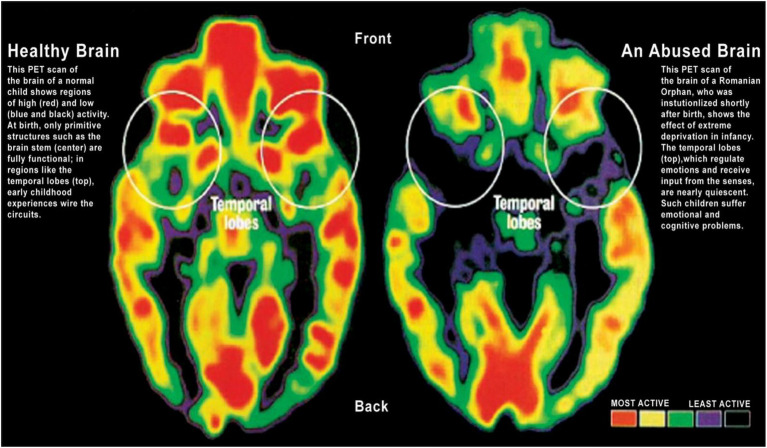
The effect of toxic stress on the healthy brain. The PET scan of the brain activity of a normal, healthy brain shows regions of high activity (red) and low activity (blue and black). In the abused brain under toxic stress, there is a significant decrease in activity in the temporal lobes, which regulate emotions. Adapted with permission from Chungani (92).

Nurturing, loving, supportive and caring environments are powerful factors in child development and positive neuroplasticity (82–86). The ‘serve and return’ reciprocal interaction between children and their parents has been shown to be effective in “brain building” as early as infancy. The model, developed by Harvard University (87), uses a tennis analogy where an infant serves (focuses his or her attention on an object), and the parent returns the serve (sharing the child’s attention and building on this). Learning continues in a supportive and encouraging environment as the child plays, develops language skills, and gains an understanding of the context and meaning of the world around them. In the absence of responsive caregiving — or if responses are unreliable or inappropriate — brain architecture does not develop as expected. It is easier to form strong neural networks in early childhood than it is to intervene or “fix” them later (88, 89). Adverse Childhood Experiences (ACE) and “toxic stress” such as exposure to domestic violence, emotional abuse, physical abuse, sexual abuse, emotional neglect, and physical neglect affect physical and mental health, and substantially affect the ability to learn, school attendance, and academic attainment (83, 90, 91). Toxic stress is cumulative and results in uncontrolled pruning processes, especially in the hippocampus, neuron loss, impaired synapses, damage to neural connectivity, and poor development of the prefrontal area of the brain responsible for thinking, problem-solving, the control of behavior (Figure 6) (83, 86, 88). This is negative neuroplasticity (23, 86).

The effects on the child increase the longer exposure to the toxic environment is allowed to continue, and the risk of long-term health complications increases as the ACE score increases. For example, exposure of children to their mother’s physical abuse and their own experience of physical and emotional abuse adds up to an ACE score of 3. This is higher than an individual with an ACE score of 0 or 1. There is a direct link between the ACE score and toxic stress in children, with a significant increase in the risk of long-term physical and mental health complications (93). The effects of fear and anxiety on cognition and memory (especially declarative memory that has an emotional impact) may be mediated through glucocorticoid effects on the hippocampus (94). Stress hormones and catecholamines are implicated in the consolidation of emotion-laden memories through arousal-induced activation of noradrenergic mechanisms within the amygdala (95). Children may display obvious signs of trauma in the classroom such as aggression or falling asleep in class; but more subtle signs such as the inability to concentrate or an unwillingness to learn are less easily discerned and less often attributed to abuse. Investment in small class sizes and real facetime with individual pupils are essential to identifying the problems that affect a child’s performance in class. Tackling environments of toxic stress and child protection are global health and education imperatives (96–98).

There is a positive correlation between clean, well-maintained, calm, ordered school environments and academic performance (99–101). Cognitive performance is reduced in ‘busy’ (distracting) visual environments compared to ‘non-busy’ visual environments (99). Poor lighting in classrooms affects both children’s health and their ability to learn (100). The availability of greenspace in the learning environment is positively associated with cognitive performance. Learning outdoors, and even watching nature from the classroom, are associated with a decrease in heart rate and cortisol levels (101). Therefore, a pedagogy of love rooted in empathy for oneself, others, and nature is essential for a child’s development, as well as for the integrity, well-being, social harmony, and economic prosperity of society.

### 5.2 Nutrition and a healthy diet

**Figure 7:**
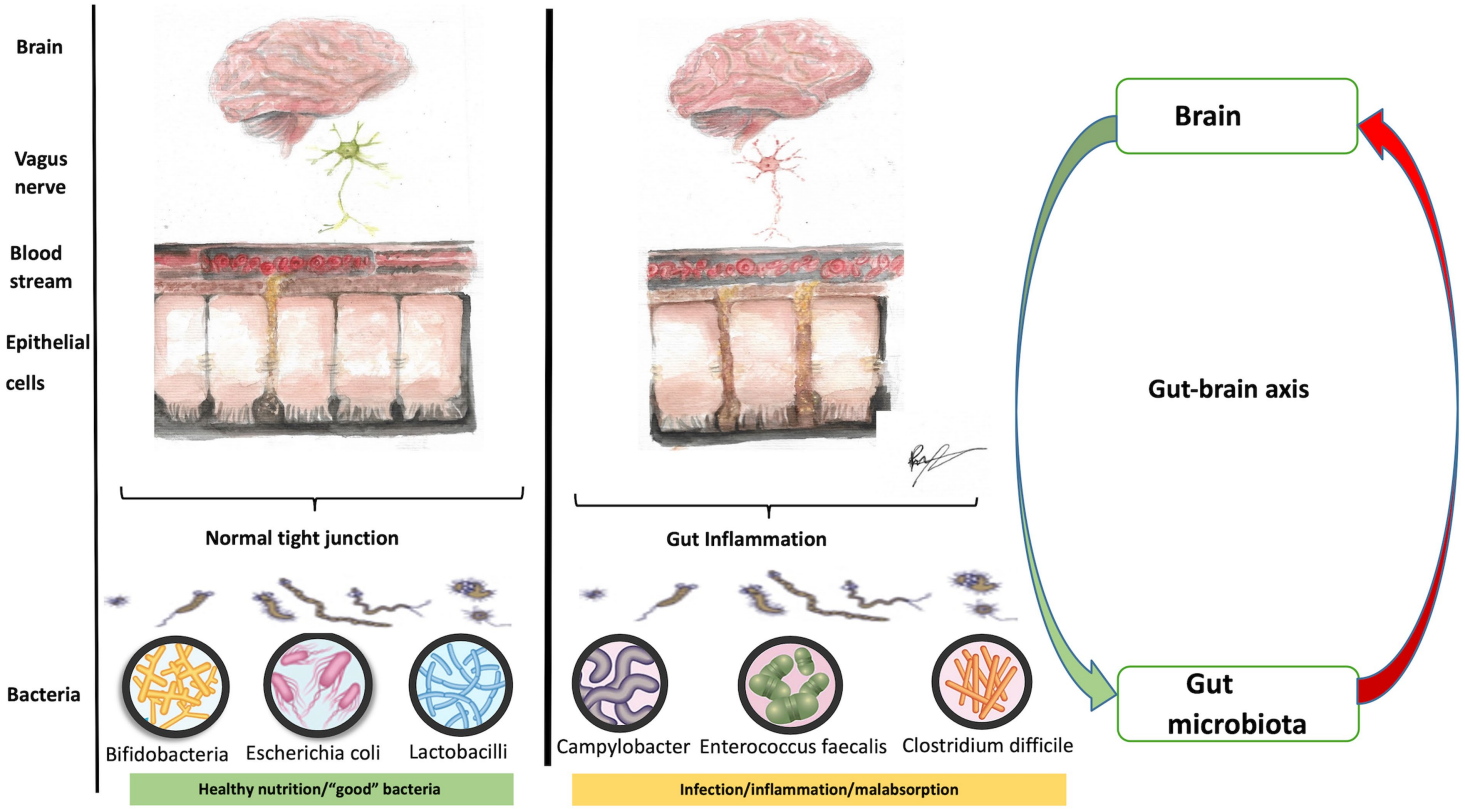
A healthy gut and the gut-brain axis (figure drawn by Roaa Mohamed).

The infant gut microbiome (the genetic material of gut microorganisms) influences neurodevelopment from birth via the gut-brain axis (Figure 7). The gut-brain axis involves the vagus nerve, immune system, hypothalamic–pituitary axis, tryptophan metabolism, and synthesis of neuroactive peptides, metabolites, short-chain fatty acids and neurotransmitters (102–111). Complete bacterial colonization is achieved within the first 3 years of life and is affected by the child’s diet, the existence of gastrointestinal disease, and exposure to antibiotics (112). A crucial function is the homeostatic mechanism of gut permeability and protection from enteric pathogens. Diarrheal diseases alone result in under-5-year mortality of up 20%, and episodes of diarrhea (three or more unformed stools per day) in the first 2 years of life may result in malnutrition-related cognitive deficits before children begin school (113).

The effects of malnutrition on brain development are profound. Chronic undernutrition and poverty in childhood are primarily measured by stunting (linear growth retardation and cumulative growth deficit). Stunted children have impaired cognition, learning, and motor function that affect school attendance, classroom participation, and learning and educational attainment (114). Fatty acids, choline, iron, zinc, cholesterol, phospholipids, and sphingomyelin play essential roles in myelination – key to white matter and cortical development (115, 116). Up to one-third of preschool children worldwide have vitamin A deficiency, 1% of whom develop night blindness (115). Nutritional inputs from infancy to school age (including breast-feeding, iron supplementation, iodine fortification, zinc, micronutrient and vitamin supplementation, and protein-energy supplementation) in community-based programs have had some success. The role school meals play in the nutrition of school age children is crucial to cognitive development and the quality of learning in schools - Learning Adjusted Years of Schooling (LAYS) (116–118). The cost of providing school meals for approximately 70 million vulnerable children is an average of $ 64 per child per year (119).

### 5.3 Physical activity and socialization

Development of the brain is affected by movement and exercise (77, 120–122). Effortless movement for only 10 min has been shown to improve the ability to remember and concentrate at all ages - from kindergarten to university (77). Ten minute breaks in class for gentle exercise may significantly improve classroom learning and performance in examinations, and are a relatively low-cost and easy intervention in the school timetable (77). Improvement in key educational competencies, especially higher orders of cognition in Bloom’s taxonomy of critical thinking, has been demonstrated after small intervals of aerobic exercise (77, 120, 121). The mechanism may be related to the relationship between movement and the secretion of BDNF. Increased BDNF secretion increases the production of mRNA which produces neurotropin, proteins, and neurotransmitters, such as dopamine, which increase anterior hippocampal volume, improve mood, motivate learning, and improve spatial memory (122, 123).

Physical activity improves cognition, increases brain volume in children with cerebral palsy, and improves phonemic skills (122). In students with visual impairment, the relationship between cognitive function and physical activity, especially in adolescents with disabilities, suggests that moderate-intensity exercise is important for brain plasticity at this age (122). Exercise may be important in developing resilience and dealing with stress (124, 125). Children who exercise as a means of coping with pressure at school should have access to sporting facilities in school that are safe, supervised, and accessible after the school day.

Prolonged periods of sitting are associated with the digital age. While unhealthy in terms of a lack of physical activity, they are also associated with other dangers, detailed discussion of which is beyond the scope of this article. Violent video game content has been shown to increase aggression, violence, depression, lack of empathy and the spectator phenomenon (126, 127). Prolonged sitting, especially in front of LED screens, leads to fatigue, disrupts sleep, biological rhythms, and reduces cognitive function (128–132). An over-reliance on screen-based learning in the classroom at the expense of writing, drawing, hands-on tasks, outdoor learning, play, and movement, especially where there is physical and social interaction between the teacher and pupils and among the pupils, is to be discouraged. Screen time is recommended to be limited to 90 min in total during the school day (133).

### 5.4 Music

**Figure 8:**
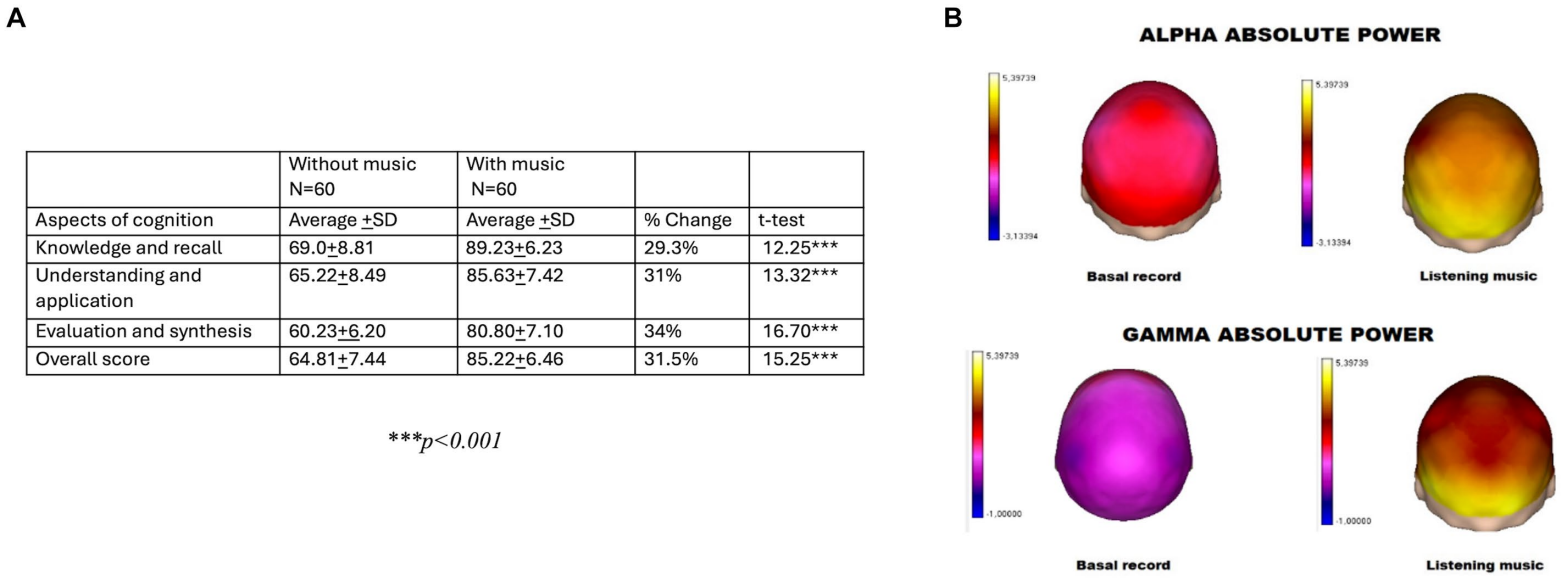
**(A)** Levels of cognition and academic performance with and without music (scale from 0–100) (78). **(B)** Changes in brain activity when listening to music (absolute power from alpha and gamma band) (78). Adapted with permission from (78), licensed under CC BY.

The positive impact of music on cognitive development, including fetal development, has been extensively studied (78, 134). Listening to Mozart has been found to significantly help mothers cope with stress and improve their temperament (134–138) which, in turn, may positively impact the child’s home environment. Music also encourages calm and restful sleep (134, 136, 139). Music is an important stimulus for the growth of functional neural networks throughout the brain in the first 3 years of childhood (6, 134–140). The effect on brain development remains significant in school-age children and is, therefore, of considerable interest in improving learning conditions in the classroom, with evidence of improvement in spatial intelligence of up to 43% among students learning to play the piano versus 11% among students studying computer science without music (136). Music also impacts intellectual development, in particular the ability to listen to and absorb language (138). There is a debate as to whether these effects are only in the short term (with no lasting effect on intelligence) but positive effects on academic achievement associated with music may be seen in the teenage years (134, 137, 139). Relatively short periods of music training have strong implications on brain plasticity (141) and have strong implications for promoting the development of music-based correction strategies for children with language-based learning disabilities (142). Further, training children in music leads to a long-term improvement in visual spatial, verbal, and mathematical performance (143). Music skills also enhance language development, literacy, literature, intelligence metrics, creativity, fine motor coordination, concentration, self-confidence, emotional sensitivity, social skills, teamwork, self-discipline, general achievement, and relaxation. Early exposure to music improves personal and social development within the context of a fun and rewarding experience (144, 145). The effects on the limbic system of pleasure and enjoyment are important motivators of learning. Playing music to children for 10 min has been shown to increase gamma waves involved in thought processes and alpha waves representing a feeling of calm (78) (Figure 8). At least 6 months of musical training in primary school is required to significantly improve behavior and influence the development of neural processes reflected in specific brain wave patterns (146, 147).

### 5.5 Sleep

**Figure 9:**
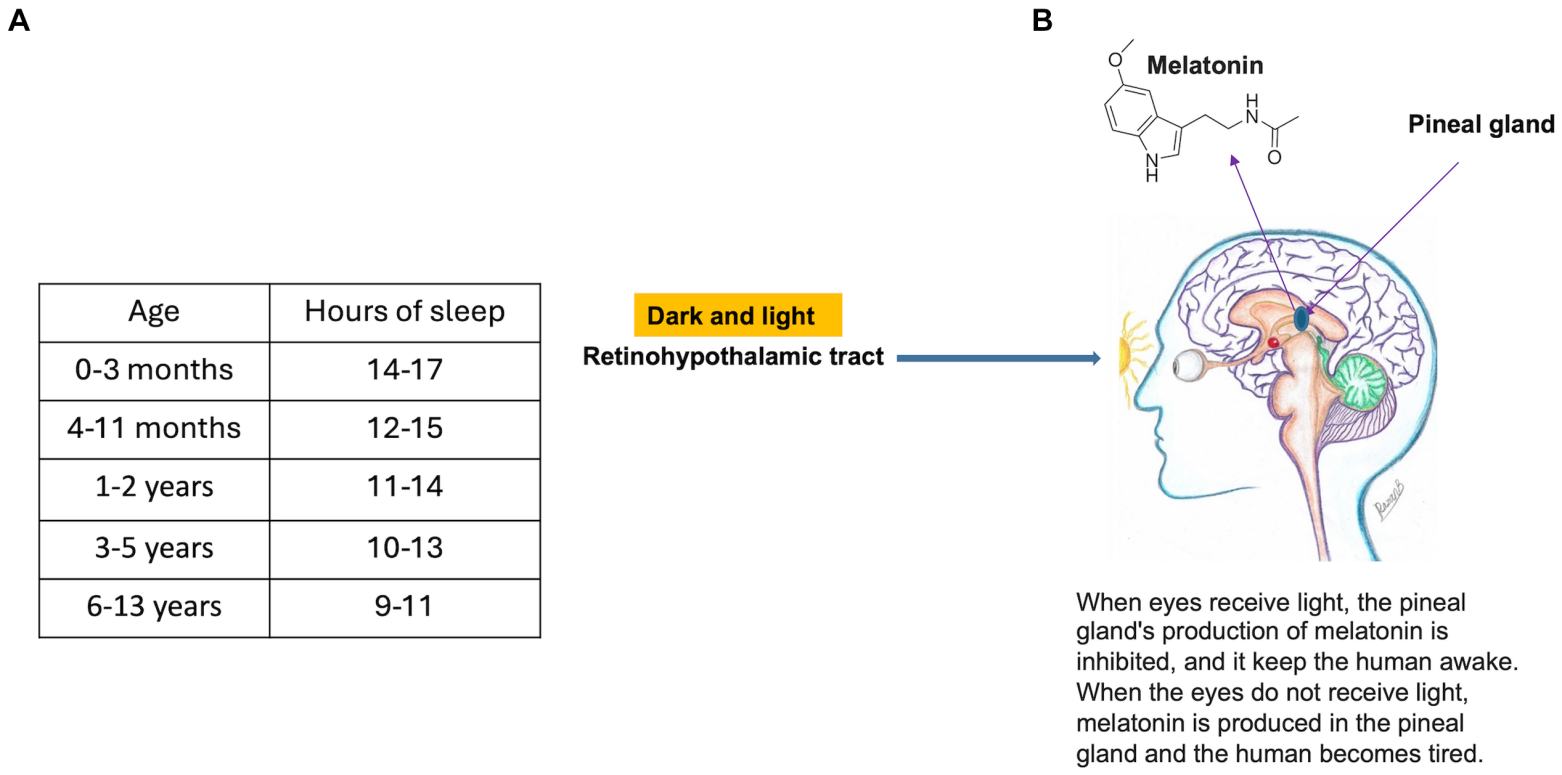
**(A)** Recommended hours of sleep based on childhood age (Data Source: 151). **(B)** Mechanism of inhibition of melatonin secretion from the pineal gland (figure drawn by Razan Bakir).

Sleep cycles begin in the womb at 23 weeks of gestation. In infancy, while the number of new neural connections formed is very high, more hours of sleep are needed for pruning and consolidation processes by which recent memories become crystallized into long-term memories (148–151) (Figure 9). By school age, the establishment of a sleep schedule becomes important. Rapid eye movement (REM) sleep is essential for the processes of short-term memory (148, 152–154). Sleep that includes REM, as well as non-REM states, is crucial to synaptic development, the support of cognitive functions, memory and plasticity (memory encoding, unification and reunion) (155–157). The process of pruning during sleep converts short-term to long-term memories. Pruning involves the clearance of amyloid, an insoluble protein precipitate, formed after the development of synapses by glial cells (148, 149). The restorative function of sleep may result from the removal of potentially neurotoxic waste products that accumulate in the central nervous system (158).

Non-REM sleep may be seen on electroencephalogram in the form of sleep spindles and K-complexes. Functional magnetic resonance imaging shows thalamic and limbic system activity during sleep, indicating their role in memory consolidation. Variations in brain activity during sleep are associated with fluctuations in cerebral oxygen demand and perfusion (159, 160).

Sleep deprivation reduces alertness, reduces the motivation to learn, limits concentration and memory formation, and affects mood (161); indeed, sometimes children fall asleep in class. Scheduled naps during the school day in kindergarten have been shown to enhance cognition and learning (162). Keeping children awake and attentive in class is a teaching challenge at any age, but shorter lessons, interesting and stimulating learning activities, inclusion and active participation, and seating struggling children closer to the teacher (or in the teacher’s eyeline) are key strategies to improve learning. The identification of problems at home, anxiety or night terrors that may result in sleep deprivation is crucial.

Melatonin is secreted by the pineal gland in the evening and night. Its secretion is stimulated by darkness and inhibited by light along the retino-hypothalamic tract (Figure 9). Infants have the highest levels, and, as a child grows, melatonin levels reduce, and secretion becomes delayed (163). Melatonin may promote deeper sleep, leading to better memory consolidation (164). Metabolites of melatonin are involved in DNA repair and free radical scavenging (165, 166). Supplementation may be effective for children with sleep disorders.

### 5.6 Brain connectivity – Gardner’s theory of multiple intelligences

**Figure 10:**
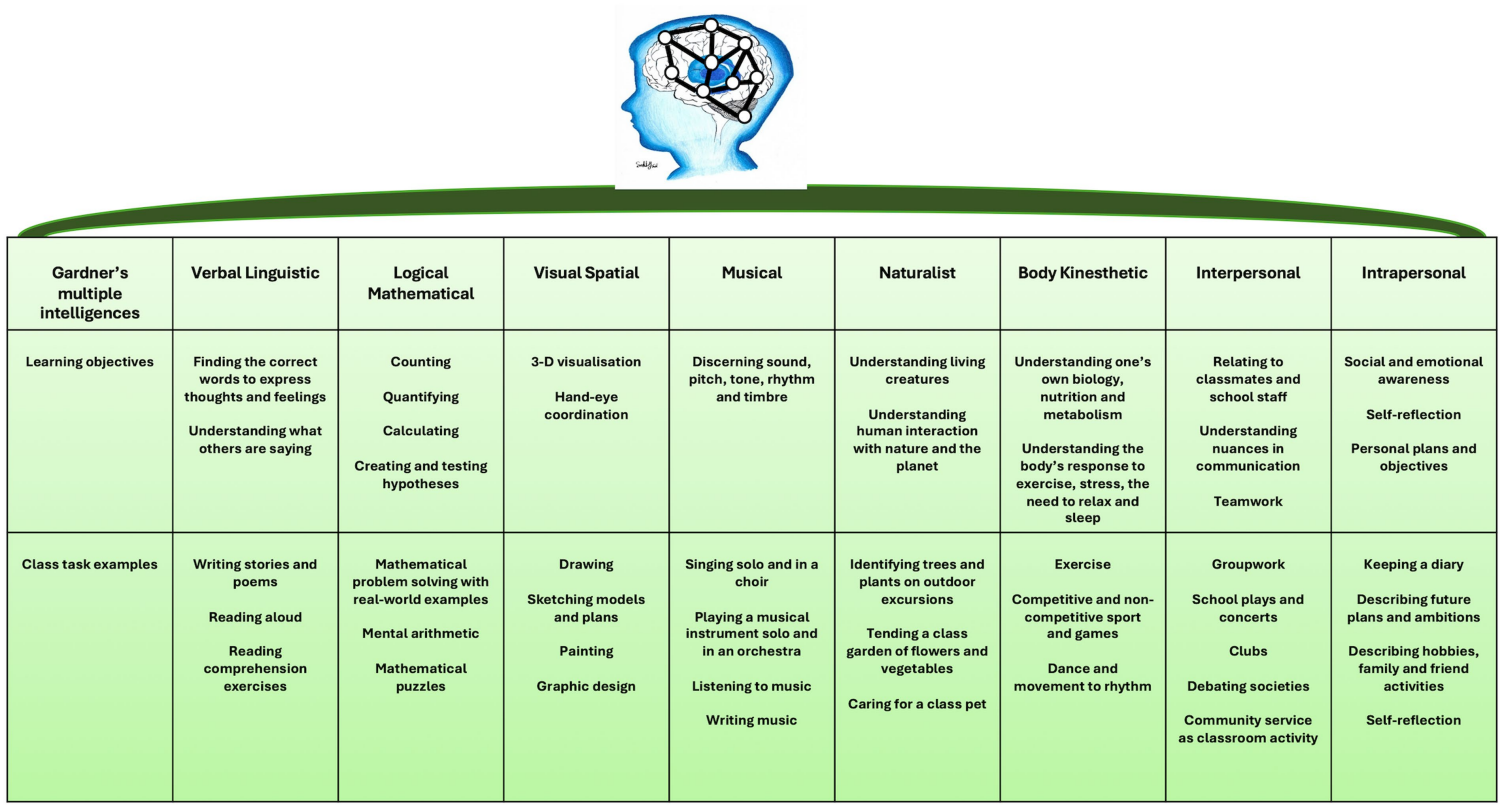
Neural networking (brain connectivity) illustrated by Gardner’s multiple intelligences in the classroom.

Intellectual potential is determined by the optimization of connections in the brain and the activation of multiple areas of the brain. Gardner’s theory of multiple intelligences sheds light on the importance of connectivity between areas of the brain and the importance of connectivity in learning (Figure 10).

While Gardner describes the existence of multiple intelligences which include verbal and linguistic, logical and mathematical, visual and spatial, musical, naturalist, body kinesthetic, interpersonal, and intrapersonal, it is crucial to understand that these diverse abilities contribute to the creation of a unified sense of self (167, 168). Gardner demonstrated that when one area of the brain is activated, another area of the brain is affected. Improvement in one particular area of the brain that expresses a particular intelligence affects the other intelligences, i.e., logical-mathematical intelligence can be improved through musical intelligence (169–171). The synthesis of various brain systems, such as the frontoparietal network, limbic system, default mode network, and attentional networks, results in the cohesive perception of being ‘one person.’ This unified experience is similar to how different chemical senses (olfaction, taste, trigeminal sensitivity) collectively create the perception of the ‘aroma’ of food, where the contribution of each sense is indistinguishable from the whole percept. Therefore, while Gardner’s model is useful for organizing educational activities (e.g., music, physical education, literature), it is essential to balance these activities to foster the development of a harmonious and integrated individual.

## 6 Using econeurobiology to tackle the social determinants of health

**Figure 11:**
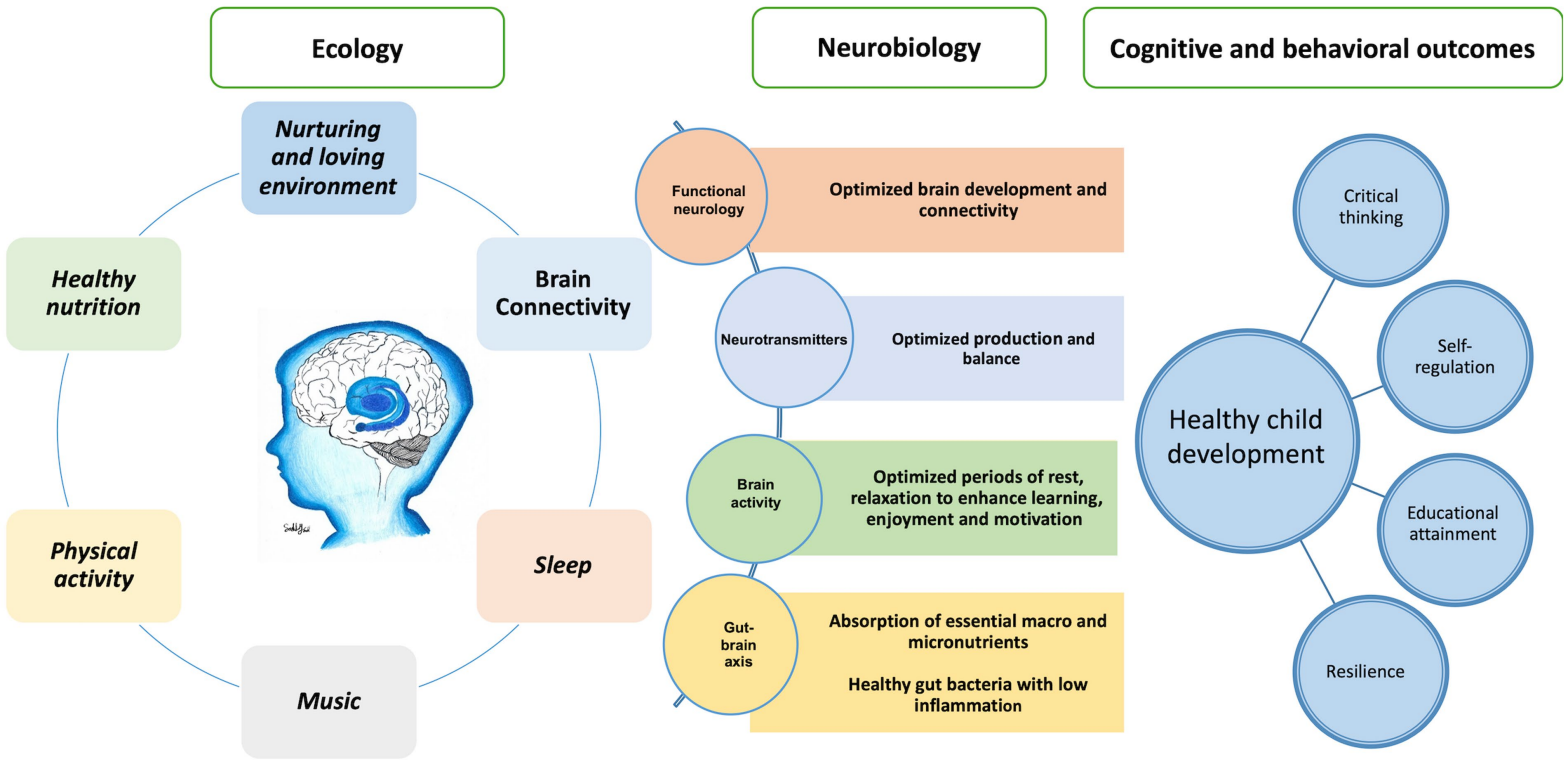
The econeurobiology of the brain for healthy child development.

We propose a model that describes the impact of a child’s ecological environment on neurological function – the ‘econeurobiology’ of brain development – Figure 11. The model refers to the ecological environment in which a child grows and the factors that shape cognition and social and emotional learning in early childhood. The model should be considered in tackling the social determinants of health, education, and child development, and creating effective and supportive environments for learning toward realization of the intellectual potential of individuals and the human capital of communities. Education lifts children out of poverty – “The fight against poverty starts with quality education for every child” (172). Music, exercise, rest and quality sleep, healthy food, and calm, ordered and nurturing environments are important in the preschool and elementary school curriculum to foster critical thinking, socialization and behavior that builds human capital.

## 7 Application of Gardner’s intelligence model, economic biology strategies, and connection to the environment of school curriculum

It is important to consider that the application of Gardner’s intelligence model and economic biology strategies may vary significantly across different cultural and institutional contexts. For example, educational policies and economic resources in the United States differ greatly from those in developing countries. Therefore, while our model provides a general framework, educators and policymakers should adapt these strategies to fit the specific needs and circumstances of their countries. This includes acknowledging the diverse educational challenges and opportunities that arise from varying economic, social, and cultural backgrounds.

Integrating Gardner’s Multiple Intelligences model into educational curricula has been shown to effectively cater to diverse student learning styles. By acknowledging students’ dominant intelligences, educators can apply Gardner’s theory across all learning types, not just those traditionally emphasized, such as verbal–linguistic and logical-mathematical intelligences (173). This approach facilitates an all-encompassing educational experience that acknowledges the significance of teachers understanding and analyzing the intelligences their students possess. The aim is to enhance learning outcomes by adopting a Multiple Intelligences-based approach tailored to each child’s unique abilities, thus improving learning achievement (173).

The educational impact of Gardner’s theory is also apparent in its ability to enrich student learning experiences at the upper elementary level (174). Analysis of its application revealed improvements in student capabilities, enabling deeper analysis and connection with previous knowledge — essential for constructing meaningful learning. By addressing the diverse learning styles and preferences, the use of multiple intelligences in the classroom promotes a more inclusive and productive educational environment (174).

When teaching strategies are tailored to the assessed multiple intelligences of students, educators are better equipped to meet the diverse needs of their classrooms, which, in turn, promotes greater academic engagement (175). Students have shown enhanced involvement and motivation when their intellectual strengths are the focus of instruction. This tailored approach not only facilitates comprehension but can also positively influence academic performance. Further studies examining the correlation between students’ achievements and their predominant intelligences could lend more credibility to these findings (175).

In summary, the integration of Gardner’s theory into educational practices allows for a nuanced approach to teaching. By adopting strategies sensitive to individual differences, educators can enhance student understanding and performance, particularly in complex areas such as physics (176).

How can econeurobiology and what we know about the psychology of cognition and learning be applied in the classroom to improve educational attainment and foster behaviors that shape healthy communities? Addressing investment in education and teaching is crucial. Under-resourced and overworked teachers would struggle to motivate and stimulate children using the best of learning and psychological strategies. Education should be seen within the context of community building and a global strategy toward prosperous and cohesive societies. Investment in teaching should be commensurate with the importance of successful child development. The importance of quality teaching and investment in school in early and mid-childhood for all children should be emphasized over the prevailing focus on higher education for the select few. Education strategies must identify and prioritize the social determinants of learning in children and optimize learning in early childhood when brain plasticity is maximal. Creating a positive and safe environment in school and addressing problems at home are fundamental. Healthy, affordable school meals that address genuine nutrition needs (vitamin A and iodine deficiency, for example) should be available to children who are malnourished.

**Figure 12:**
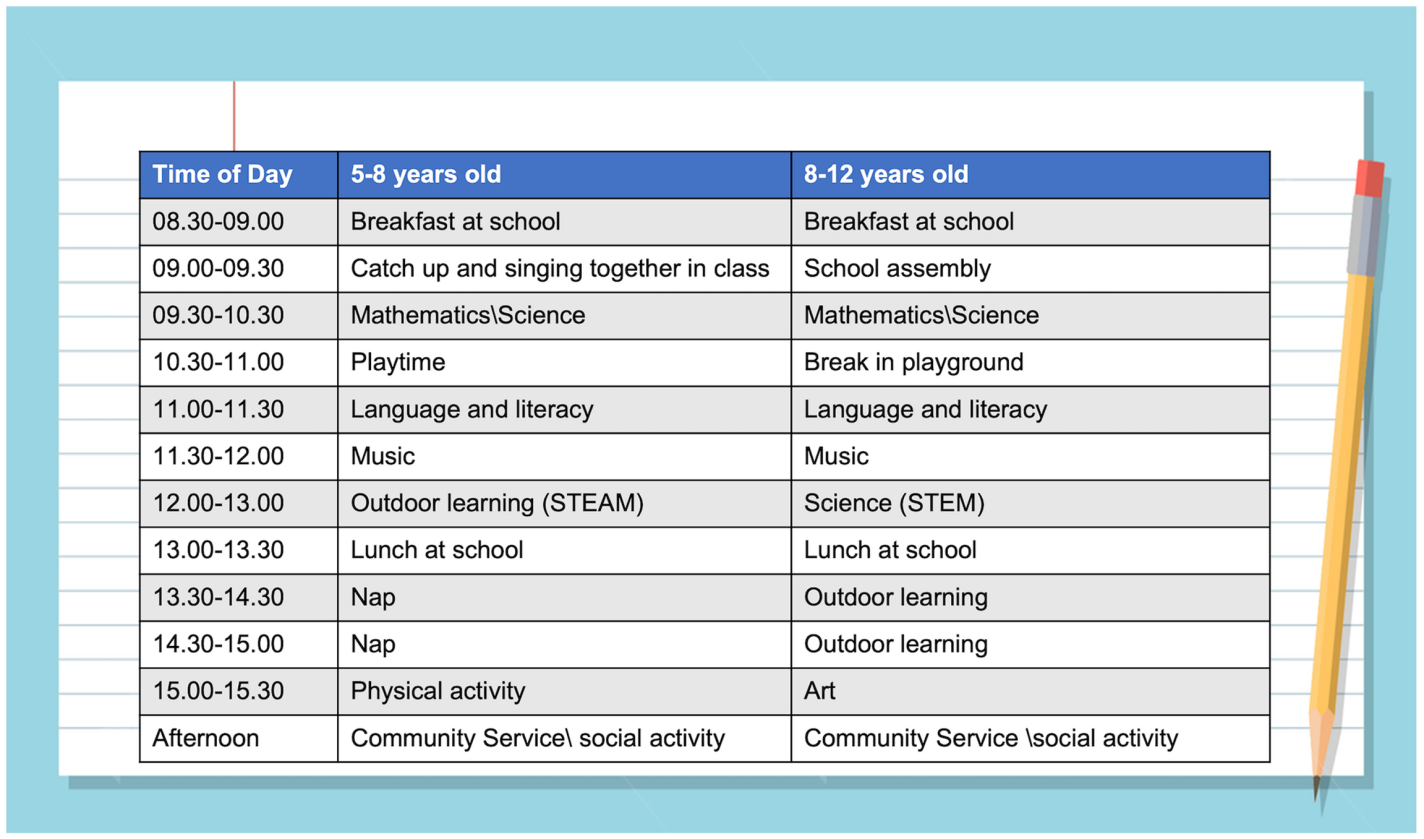
Optimizing the elementary school schedule – a typical day for children aged 5 and 10 years (a combination of stimulation and relaxation to optimize learning).

Ten minutes of relaxing music or aerobic exercise before class can prime children for their lessons (Figure 12). In class, four strategies have been found to stimulate learning and promote memory formation (177, 178): (1) retrieval practice (questioning pupils to elicit their recall and retrieval of information rather than lecturing to passive listeners); (2) feedback (pupils become self-aware of what they know and understand and the gaps in their learning). This stimulates and focuses new learning and increases depth of understanding; (3) spaced-practice (knowledge and understanding consolidated in stages over time so that pupils can process, reorganize and apply what they have learned). This facilitates the formation of lasting memories; (4) interleaving (acquiring new information through a variety of teaching and learning methods – a mix of skills that stimulate brain connectivity and memory formation).

## 8 The importance of science, technology, engineering, art and mathematics

STEAM education plays a vital role in preparing students for the future, but its implementation can be influenced by cultural and economic factors. For instance, the emphasis on different subjects within STEAM may vary depending on national priorities and resources. In some countries, there might be a greater focus on technology and engineering due to industrial needs, while others might prioritize science and mathematics based on educational traditions. Additionally, the availability of resources for hands-on learning and extracurricular activities can differ, affecting the overall effectiveness of STEAM programs. By considering these cultural and economic variations, we can better tailor STEAM education to meet the unique needs of students globally.

Children have fallen behind in science, technology, engineering, art and mathematics (STEAM) education, in reading, and in literacy (179, 180). We now have a reduced adult STEAM workforce, reduced adult literacy and reduced engagement with book reading (181). Literacy, science, and mathematical skills are essential to industrialization, a productive workforce, and the economic prosperity of nations. Children from vulnerable backgrounds are particularly disadvantaged and less likely to pursue STEAM subjects in higher education (182). Yet, effective teaching of STEAM subjects can stimulate and fire the imagination of children. These subjects lend themselves to practical teaching strategies that translate theories into tangible and real experiments, drama classes, drawing, and model-making that are fun and engaging – enhancing brain connectivity for optimal learning. They introduce real-world examples into the classroom where the relevance of STEAM concepts are obvious and learning is translational and modular. Theoretical concepts that are time-consuming, boring, laborious and difficult to explain become practical problem-solving exercises, explorations and analyses of everyday (real-life) activities that interest motivate and stimulate learning – augmenting connectivity between cortical and social and emotional learning centers from an early age.

Scientific and technological literacy are the basic tools and strategies employed in research and discovery. They develop skills of critical thinking and enable students to generate new knowledge. Education of STEAM subjects is crucial to the overall development of the brain architecture, specifically in the prefrontal cortices and the strengthening of top-down self-regulation pathways. Through STEAM education, students learn how to think, rather than being taught what to think - promoting independent and analytical thinking.

## 9 Conclusion

Brain connectivity is engaged at all levels of Bloom’s taxonomy of critical thinking, both simultaneously and cumulatively. Active inquiry-based learning strategies surpass traditional passive didactic methods in enhancing brain connectivity, leading to more effective educational outcomes. Contextual learning strategies, particularly those incorporating real-world examples and outdoor experiences, serve to bolster cognition and memory. They add relevance and a sense of achievement, making learning more rewarding.

Competency-based educational strategies test the application of knowledge, skills, and attitudes, providing critical feedback for teachers and learners. This feedback catalyzes the acquisition of new knowledge and the reinforcement of successful behavioral models, which then evolve into essential life skills. Through such strategies, children learn the meta-skill of learning itself—a fundamental tool for lifelong education.

The relevance of school curricula to the communities they serve is paramount to ensuring that children remain engaged in their education. Extracurricular activities should complement and extend classroom learning, incorporating low-cost sports and team-building exercises into a broader curriculum framework. Opportunities such as sporting events, drama clubs, choirs, bands, debating societies, school journals, and community service projects should not be exclusive to well-funded schools. Instead, they should be leveraged to address educational determinants and meet community needs, especially in underprivileged and unstable environments (183–188).

We call upon policymakers and international institutions to recognize the critical role of designing supportive econeurobiological environments within communities and schools. Safe and nurturing settings enable children to acquire skills essential for building healthy, caring, and prosperous societies. We also call for the immediate action to remove children from war and violent conflicts, and to establish effective strategies to mitigate the negative effects of war by re-establishing healthy and functional econeurobiology.

In summary, the convergence of econeurobiology and educational strategy presents a pivotal opportunity for transformation. By leveraging insights into brain development within educational and community contexts, we can cultivate environments that not only bolster learning and cognitive growth but also contribute to forging more resilient, healthier, and united societies.
